# The Separation and Characterization of Extracellular Vesicles from Medium Conditioned by Bovine Embryos

**DOI:** 10.3390/ijms21082942

**Published:** 2020-04-22

**Authors:** Krishna Chaitanya Pavani, Xiaoyuan Lin, Joachim Hamacher, Wim Van Den Broeck, Liesbeth Couck, Luc Peelman, An Hendrix, Ann Van Soom

**Affiliations:** 1 Department of Reproduction, Obstetrics and Herd Health, Faculty of Veterinary Medicine, University of Ghent, Salisburylaan 133, B-9820 Merelbeke, Belgium; Ann.Vansoom@ugent.be; 2 Department of Nutrition, Genetics and Ethology, Faculty of Veterinary Medicine, Ghent University, B-9820 Merelbeke, Belgium; linxiaoyuan23@163.com (X.L.); Luc.Peelman@ugent.be (L.P.); 3 Plant Diseases and Crop Protection, Institute of Crop Science and Resource Conservation, University of Bonn, Nussallee 9, 53115 Bonn, Germany; hamacher@uni-bonn.de; 4 Department of Morphology-Faculty of Veterinary Medicine, Ghent University, Salisburylaan 133, B-9820 Merelbeke, Belgium; Wim.VandenBroeck@UGent.be (W.V.D.B.); liesbeth.couck@ugent.be (L.C.); 5 Laboratory of Experimental Cancer Research, Department of Human Structure and Repair, Ghent University, B-9000 Ghent, Belgium; an.hendrix@ugent.be; 6 Cancer Research Institute Ghent (CRIG), B-9000 Ghent, Belgium

**Keywords:** extracellular vesicles, isolation, protocols, ultracentrifugation, size exclusion chromatography, Optiprep^TM^ density gradient

## Abstract

Extracellular vesicles (EVs) have been identified as one of the communication mechanisms amongst embryos. They are secreted into the embryo culture medium and, as such, represent a source of novel biomarkers for identifying the quality of cells and embryos. However, only small amounts of embryo-conditioned medium are available, which represents a challenge for EV enrichment. Our aim is to assess the suitability of different EV separation methods to retrieve EVs with high specificity and sufficient efficiency. Bovine embryo-conditioned medium was subjected to differential ultracentrifugation (DU), OptiPrep^TM^ density gradient (ODG) centrifugation, and size exclusion chromatography. Separated EVs were characterized by complementary characterization methods, including Western blot, electron microscopy, and nanoparticle tracking analysis, to assess the efficiency and specificity. OptiPrep^TM^ density gradient centrifugation outperformed DU and SEC in terms of specificity by substantial removal of contaminating proteins such as ribonucleoprotein complexes (Argonaute-2 (AGO-2)) and lipoproteins (ApoA-I) from bovine embryo-derived EVs (density: 1.02–1.04, 1.20–1.23 g/mL, respectively). In conclusion, ODG centrifugation is the preferred method for identifying EV-enriched components and for improving our understanding of EV function in embryo quality and development.

## 1. Introduction

Over the last two decades, extracellular vesicles (EVs) have been recognized as a critical component of intercellular communication in both physiological and pathological conditions [1]. Interestingly, they are present in all kinds of biological fluids and contain a myriad of biomolecules, such as proteins, nucleic acids, and metabolites. Therefore EVs represent a very attractive source of potential biomarkers, particularly in the enhancement of assisted reproductive technologies (ART) in humans.

Several sophisticated models based on multivariate parameters of embryo scoring have been designed to predict pregnancy rates in humans after in vitro fertilization and to optimize the selection of embryos in specific subgroups of patients [2]. Although these models are a step forward, they still rely on morphological observations of embryo development and do not take into account the genetic constitution of the embryo. Embryos carrying chromosomal abnormalities will have a lower chance of implantation or will lead to a higher percentage of pregnancy loss after implantation [3,4].

Embryonic secretions might predict the true developmental capacity of an embryo. The embryo culture medium could, therefore, reveal important quality aspects of the embryo that could assist the selection of the most competent embryos [5]. Recently, our group has demonstrated that bovine embryos cultured in a group can release functionally active EVs with sizes ranging from 25 to 250 nm. Embryo-derived EVs are possible autocrine embryotropins playing a crucial role in embryo development [6]. Recently, Giacomini et al. [7] have also shown that conditioned media from human embryos cultured in vitro for 3 days or up to the blastocyst stage contained EVs with a diameter of 50 to 200 nm.

The separation of EVs still represents a critical step due to several reasons: first, the methods to isolate EVs are currently highly diverse, and second, depending on which separation method is employed, the results can be considerably different, even when starting from the same sample [8]. To date, DU is the most popular technique for the separation of EVs, which typically consists of low-speed centrifugation to remove cells and large vesicles followed by high-speed ultracentrifugation to pellet EVs [9]. This method relies on sedimentation at high speed for separating EVs from other (extra)cellular components [10,11]. Density gradient-based centrifugation, using sucrose or iodixanol (OptiPrep^TM^), can be utilized to remove contaminating impurities such as non-specific argonaute proteins, and high-density lipoproteins [8]. Apart from both ultracentrifugation methods, size exclusion chromatography (SEC) is also commonly used. Size-exclusion chromatography separates EVs from other media components based on their size, whereby particles with different sizes move through the filtration column at different rates.

Our aim is to assess the suitability of different methods to separate EVs with maximum specificity and efficiency from embryo-conditioned medium. We evaluate the impact of three separation methods (differential ultracentrifugation (DU), OptiPrep™ density gradient ultracentrifugation (ODG), and size-exclusion chromatography (SEC)) on yield, purity, size, and morphology of EVs secreted by bovine embryos.

## 2. Results

**Figure 1:**
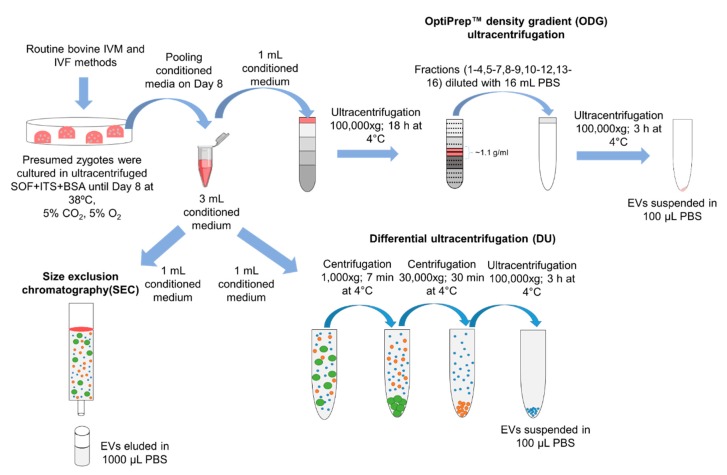
Schematic overview of three methods to separate EVs from bovine embryo-conditioned medium. A total of 1500 presumed zygotes were cultured in groups of 25 in 50 µL droplets of ultracentrifuged synthetic oviductal fluid enriched with non-essential and essential amino acids (SOFaa), ITS (5 µg/mL insulin; 5 µg/mL transferrin; 5 ng/mL selenium) and medium droplets were covered with mineral oil and incubated at 38 °C in 5% CO_2_, 5% O_2_, and 90% N_2_. After 8 days of post insemination, the bovine embryo-conditioned medium was pooled and equally divided over three separation methods: differential ultracentrifugation (DU), OptiPrep^TM^ density gradient centrifugation (ODG), and size exclusion chromatography (SEC).

A total of 1500 presumed zygotes were cultured in ultracentrifuged SOF + ITS + BSA to attain 3 mL of conditioned medium on Day 8. For each EV separation method, 1 mL of conditioned medium was used. A schematic representation of the DU, ODG, and SEC isolation protocols used in this study is shown in Figure 1.

### 2.1. Samples Separated by Differential Ultracentrifugation Contain Contaminants and Aggregates of Extracellular Vesicles

**Figure 2:**
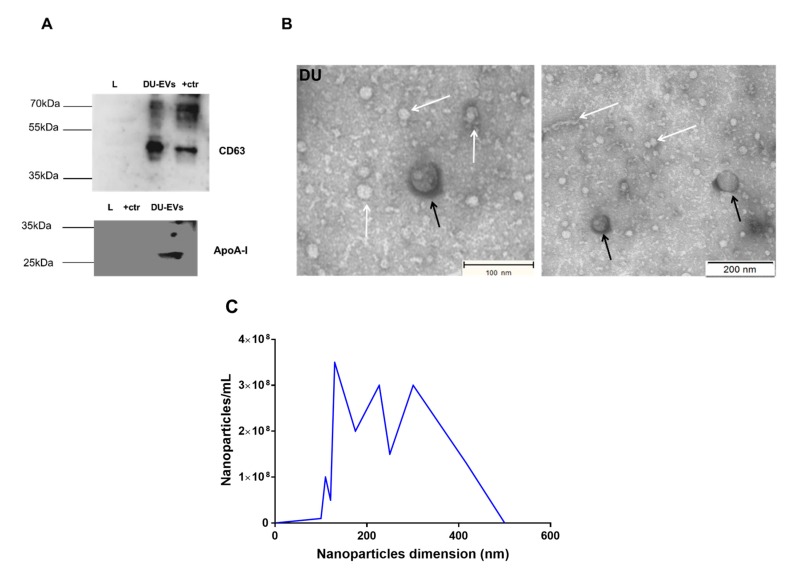
Characterization of DU-EVs derived from bovine embryo-conditioned medium. (**A**) Western blot analysis of EV-associated protein CD63 (42 kDa) and non-EV associated proteins (ApoA-I (28 kDa)). (**B**) Transmission electron microscopy pictures of DU-EVs with white arrows representing protein aggregates and black arrows indicating EVs. Scale bar = 100/200 nm. (**C**) Size distribution profile of DU-EVs determined by NTA. Abbreviations: DU = differential ultracentrifugation; +ctr = follicular fluid derived EVs by OptiPrep^TM^ density gradient; L = marker (protein ladder).

Western blot analysis of EV preparations obtained by differential ultracentrifugation (DU) revealed the presence of EV-associated protein CD63 (Figure 2A). The DU pellet also contained high-density lipoprotein (HDL) marker apolipoprotein A-I (ApoA-I), and argonaute-2 (AGO-2) protein, a member of the RNA-induced silencing complex and a well-characterized extracellular RNA-binding protein (Figure 2A), indicating contamination of EVs with other extracellular particles. To cross-check the specificity of the Western blot bands, additional Western blotting was performed with incubation of secondary antibodies only (anti-rabbit IgG with 5% BSA for CD63 and anti-mouse IgG with 5% milk for ApoA-I and AGO-2). We did not observe any non-specific bands with regards to the antibodies CD63 (42 kDa), AGO-2 (97 kDa), and ApoA-I (28 kDa), proving the specificity of the primary antibody (see Figure S1). TEM analysis confirmed the presence of EVs of approximately 100 to 260 nm in size (Figure 2B). In addition, TEM demonstrated the presence of protein aggregates and lipoprotein particles (Figure 2B). Next, we quantified the size and concentration of particles in DU preparations by NTA (Figure 2C). The particle concentration starting from a pool of 500 embryos was 3.02 ± 0.36 × 10^10^ particles/mL, and the mean and mode particle size were 266.8 ± 2.1 and 186.2 ± 21.9 nm, respectively (as detailed in Table 1, Figure 2C).

### 2.2. Size Exclusion Chromatography does not Separate Protein Aggregates and Lipoprotein Particles from Extracellular Vesicles

**Figure 3:**
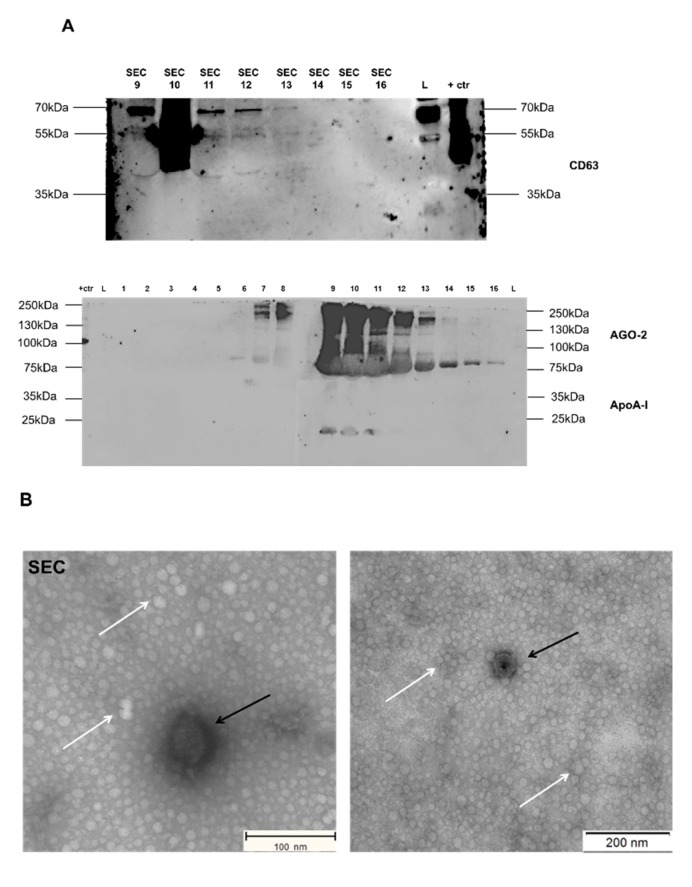

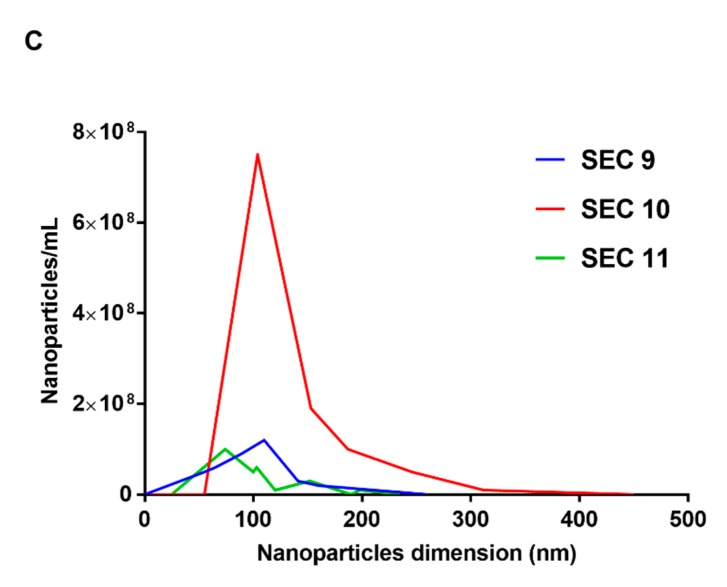
Characterization of SEC-EVs derived from bovine embryo-conditioned medium. (**A**) Western blot analysis of EV-associated protein CD63 (42 kDa) and non-EV associated proteins (ApoA-I (28 kDa), Ago-2 (97 kDa)). (**B**) Transmission electron microscopy pictures of SEC-EVs with white arrows representing protein aggregates and black arrows indicating EVs. Scale bar = 100/200 nm. (**C**) Size distribution profile of SEC-EVs determined by NTA. Abbreviations: SEC 1 = size exclusion chromatography fraction 1 (similar for 2 to 16 fractions); +ctr = follicular fluid derived EVs by OptiPrep^TM^ density gradient; L = marker (protein ladder).

Western blot analysis identified EV-associated protein CD63 across fractions 9 to 11. In addition, the majority of argonaute-2 (AGO-2) protein and apolipoprotein A-I (ApoA-I) was detected in fractions 9 to 11 (Figure 3A). Additional Western blotting analysis with secondary antibody (anti-rabbit IgG with 5% BSA for CD63 and anti-mouse IgG with 5% milk for ApoA-I, AGO-2) incubation showed some non-specific bands in the fractions of SEC 1 to 8 and SEC 10 (see Figure S2). No bands were observed with regards to ApoA-I (28 kDa), AGO-2 (97 kDa), and CD63 (42 kDa) from fractions 1 to 8 and fraction 10, proving the specificity of the primary antibody (Figure 3A). TEM analysis of fraction 10 confirmed the presence of EVs approximately 25 to 250 nm in size and protein aggregates (Figure 3B). SEC fraction 10 showed the highest particle concentration with 1.50 ± 0.43 × 10^11^ particles/mL starting from a pool of 500 embryos with a mean particle size of 119.2 ± 3.5 nm and a mode of 99.7 ± 6.0 nm as measured by NTA (Table 1, Figure 3C).

### 2.3. OptiPrep^TM^ Density Gradient Centrifugation Separates Extracellular Vesicles with Higher Specificity Compared to DU and SEC

**Figure 4:**
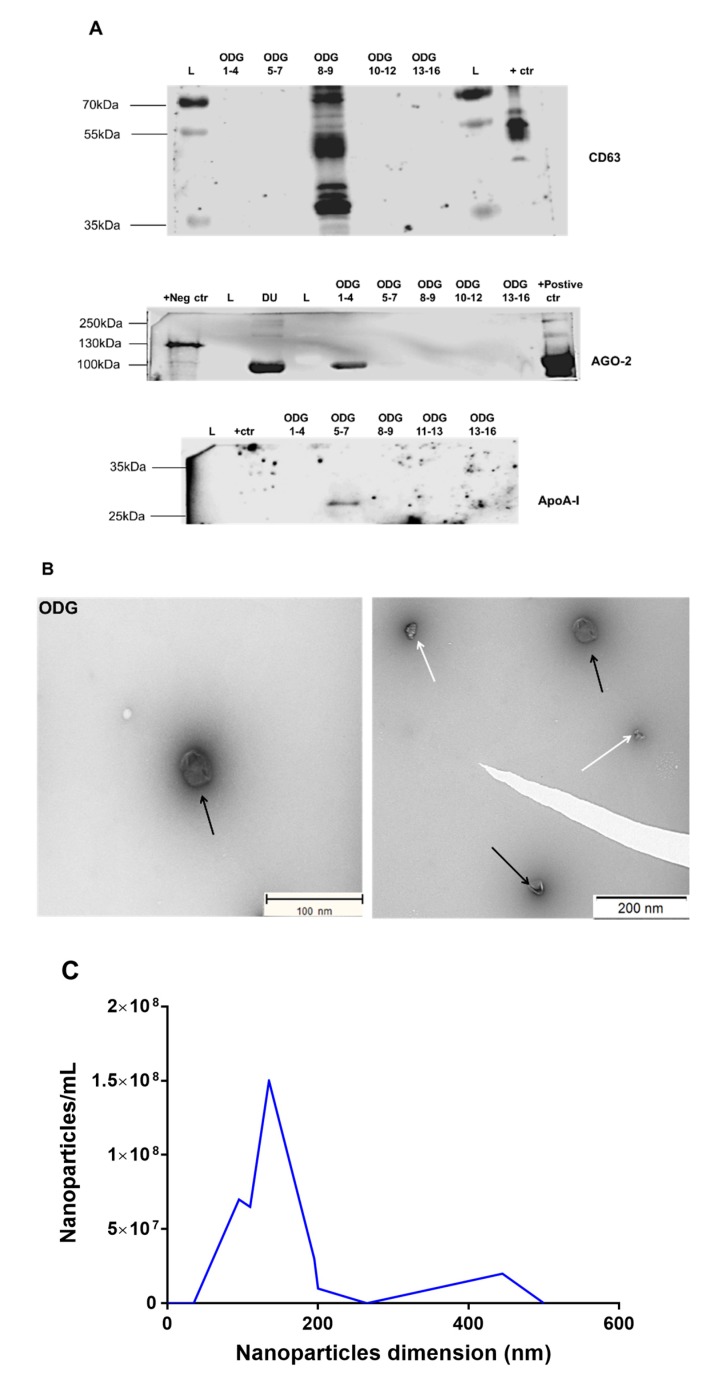
Characterization of ODG-EVs derived from bovine embryo-conditioned medium. (**A**) Western blot analysis of EV-associated protein CD63 (42 kDa) and non-EV associated proteins (ApoA-I (28 kDa), Ago-2 (97 kDa)). (**B**) Transmission electron microscopy pictures of ODG-EVs with white arrows representing protein aggregates and black arrows indicating EVs. Scale bar = 100/200 nm. (**C**) Size distribution profile of ODG-EVs determined by NTA. Abbreviations: DU = differential ultracentrifugation; ODG 1–4 = OptiPrep^TM^ density gradient fractions 1 to 4 (similar for 5–7, 8–9. 10–12, 13–16); +ctr = follicular fluid derived EVs by ODG, L = marker (protein ladder), +Neg ctr = EVs derived from Lysate HEK 293T, +Positive ctr = Plasma pure.

Next, we analyzed the specificity of ODG centrifugation for enriching EVs from bovine embryo-conditioned medium. Western blot analysis identified EV-associated protein CD63 density fractions 8 and 9, corresponding to a density of 1.086–1.119 g/mL (Figure 4A). By contrast, the majority of argonaute-2 (AGO-2) protein and apolipoprotein A-I (ApoA-I) were detected in, respectively, fractions 1 to 4 and 5 to 7 (density: 1.02–1.04, 1.20–1.23 g/mL, respectively; Figure 4A). There are some non-specific bands (~80 kDa) in the sample fraction of ODG 1–4 and a positive control sample of the secondary antibody Western blotting analysis (see Figure S1), whereas no additional bands were observed with regards to primary antibody incubation proving the specificity of AGO-2 antibody (Figure 4A). The TEM analysis of density fractions 8 and 9 confirmed the presence of EVs of approximately 25 to 250 nm in size (Figure 4B). The particle distribution assessed by NTA ranged from 25 to 300 nm, with a mean particle size of 151.3 ± 8.8 nm, mode of 100.1 ± 20.4 nm, and a particle concentration of 5.50 ± 2.70 × 10^9^ particles/mL, starting from a pool of 500 embryos (Table 1, Figure 4C).

## 3. Discussion

There is a growing need for a standardized method to prepare EVs with sufficient specificity and efficiency. Multiple studies comparing different methods for separation of EVs from biological fluids and cell culture media have been published [8,12,13]. Such a comparison for medium conditioned by embryos has not been performed yet. Here, we assessed the efficiency of different EV separation methods, including DU, ODG centrifugation, and SEC. We show that ODG centrifugation separated EVs from bovine embryo-conditioned media with higher specificity compared to DU and SEC. As has been demonstrated by previous studies [8,14], ODG depletes EV samples from ribonucleoprotein complexes (argonaute-2) and lipoprotein particles (apolipoprotein A-I (ApoA-I)). In agreement with our findings, Van Deun et al. [8] also reported the presence of AGO-2 protein in EVs obtained by DU. In addition, DU may yield EV aggregates after pelleting [10,11,15,16]. Alternatively, it may enrich for larger EVs that sediment more efficiently. ODG centrifugation separates a wide range of smaller to larger EVs with high efficiency, confirming the results by Van Deun et al. [8].

When using ODG and SEC, the vesicle structure and integrity of EVs remain largely intact, and the biological activity of EVs is preserved. The size distribution profile of SEC- and ODG-derived EVs differed from DU-derived EVs, as SEC- and ODG-derived EVs were found to be smaller in size compared to DU-derived EVs. Larger size vesicles derived by DU may be the result of aggregation or fusion of EVs during ultracentrifugation, as also suggested by others [10,17,18]. The major disadvantages of using SEC methods are the co-precipitation of other extracellular particles like lipoprotein particles and ribonucleoproteins, as demonstrated per the current study. These findings are in accordance with Takov et al. [19,20], who demonstrated that lipoproteins are co-isolated with SEC-EVs derived from plasma.

One of the major challenges in isolating EVs is the input volume of embryo culture medium. Earlier, Mellisho et al. [21] have demonstrated that 500 µL of medium conditioned by bovine blastocysts was sufficient for EV isolation, but apparently, in this study, no standardized EV isolation method was applied. In our previous study, initially, we used 500 µL of embryo-conditioned medium, but this was not sufficient for either characterization or quantification of EVs [6]. In conclusion, the separation of EVs with high specificity from 1 mL of embryo culture medium was addressed in the current study. Our analyses highlight the ODG centrifugation method as the most suitable procedure for EV enrichment that results in greater purity than DU and SEC. ODG centrifugation will allow the improved identification of EV-associated molecules that may further be used as biomarkers for embryo quality.

## 4. Material and Methods

### 4.1. In Vitro Embryo Production

Routine in vitro methods were used for bovine embryo production, as described previously by Pavani et al. [6]. Briefly, at the local abattoir, cow ovaries were obtained and processed within 2 h after collection. Upon arrival at the lab, ovaries were washed three times in warm physiological saline (with kanamycin (25 mg/mL)). Cumulus-oocyte complexes (COCs) were aspirated from antral follicles sizing between 4 and 8 mm diameter using an 18-gauge needle attached to a 10-mL syringe. Subsequently, only oocytes with uniformly granulated cytoplasm and surrounded by more than three compact layers of cumulus cells were cultured in groups of 60 COCs in 500 mL modified bicarbonate buffered TCM-199 (supplemented with 50 mg/mL gentamicin and 20 ng/mL epidermal growth factor) in 5% CO_2_ in air for 22 h at 38.5 °C.

Frozen-thawed bull spermatozoa were separated using a 45/90% Percoll^®^ gradient (GE Healthcare Biosciences, Uppsala, Sweden). Next, the sperm pellet was washed in IVF–Tyrode’s albumin–pyruvate–lactate (TALP) medium, containing bicarbonate-buffered Tyrode solution. Then, it was adjusted to a final concentration of 1 × 10^6^ spermatozoa/mL using IVF–TALP medium enriched with BSA (Sigma A8806; 6 mg/mL) and heparin (25 mg/mL).

After 22 h maturation, bovine oocytes were washed in 500 µL IVF–TALP and subsequently co-incubated with Percoll^®^ washed bull spermatozoa. After 21 h gamete co-incubation, presumed zygotes were vortexed to remove surplus zona attached cumulus and sperm cells. The presumed zygotes were cultured in groups of 25 in 50 µL droplets of ultracentrifuged synthetic oviductal fluid enriched with non-essential and essential amino acids (SOFaa), ITS (5 µg/mL insulin; 5 µg/mL transferrin; 5 ng/mL selenium) and medium droplets were covered with mineral oil and incubated at 38 °C in 5% CO_2_, 5% O_2_, and 90% N_2_.

### 4.2. Collection of Culture Media Conditioned by Bovine Embryos

Culture medium conditioned by bovine embryos was collected and pooled in preparation for EV isolation. Per embryo culture replicate, a group of 100 presumed zygotes was cultured in SOF + ITS + BSA culture medium (UC BSA medium; 25 zygotes per 50 µL droplet depleted from EVs after 100,000× *g* of ultracentrifugation for 18 h at 4 °C). On 8 dpi, 200 µL embryo-conditioned medium was collected and stored at −80 °C. To obtain 3 mL of embryo-conditioned culture medium, 15 replicates were performed with a total of 1500 presumed zygotes. One mL of pooled embryo-conditioned medium was used for each EV isolation method (differential ultracentrifugation, OptiPrep™ density gradient ultracentrifugation, and size-exclusion chromatography; as detailed in Figure 1).

### 4.3. Differential Ultracentrifugation

Differential ultracentrifugation was performed as described previously by Théry et al. [9] with a few modifications. Briefly, 1 mL of embryo-conditioned medium was diluted to 5 mL in phosphate-buffered saline (PBS) (Invitrogen), transferred to a 5.2-mL open-top polyallomer centrifuge tube (Beckman Coulter, Fullerton, CA, USA) and centrifuged for 7 min at 10,000× *g* and 4 °C in a swinging bucket centrifuge (Optima XPN-80, SW 55 Ti rotor, Beckman Coulter). The pellet was discarded, and the supernatant was centrifuged for 30 min at 30,000× *g* and 4 °C, again the pellet was discarded, and the supernatant was centrifuged for 3 h at 100,000× *g* and 4 °C. The pellet was resuspended in 100 µL of PBS and stored at −80 °C for further EV characterization.

### 4.4. OptiPrep^TM^ Density Gradient Ultracentrifugation

OptiPrep™ density gradient (ODG) ultracentrifugation was conducted, as previously reported by Van Deun et al. [8]. Briefly, appropriate amounts of a homogenization buffer (10 mM Tris-HCl (tromethamine-hydrochloric acid), 1 mM EDTA (Ethylenediaminetetraacetic acid) and 0.25 M sucrose (pH 7.4)) and an iodixanol working solution were mixed in order to prepare 5%, 10%, 20%, and 40% iodixanol solutions. The iodixanol working solution was made by adding a working solution buffer (60 mM Tris-HCl, 6 mM EDTA, 0.25 M sucrose (pH 7.4)) to a stock solution of OptiPrep™ (60% (*w/v*) aqueous iodixanol solution). Moreover, the gradient was prepared in a 16.8 mL open-top polyallomer tube (Beckman Coulter) by layering 4 mL of 40%, 4 mL of 20%, 4 mL of 10%, and 3.5 mL of 5% solutions on top of each other. One mL of embryo-conditioned medium was overlaid onto the top of the gradient. Subsequently, the gradient was centrifuged at 4 °C for 18 h at 100,000× *g* (SW 32.1 Ti rotor, Beckman Coulter). Then, all 16 gradient fractions were divided into six samples by pooling the fractions 1–4, 5–7, 8–9, 10–12, 13–16, respectively. The pooled fractions were added to 14 mL PBS. Subsequently, the separate suspensions were centrifuged at 4 °C for 3 h at 100,000× *g*. The resulting pellets were resuspended in 100 µL PBS and stored at −80 °C for further EV characterization.

### 4.5. Size-Exclusion Chromatography

Sepharose CL-2B (GE Healthcare, Uppsala, Sweden) was washed three times with PBS containing 0.32% trisodium citrate dihydrate (ChemCruz, Dallas, TX, USA) [22,23]. For the preparation of one column, nylon net with 20 μm pore size (NY2002500, Merck Millipore, Billerica, MA, USA) was placed on the bottom of a 10-mL syringe (BD Biosciences, San Jose, CA, USA), followed by stacking of 10 mL Sepharose CL-2B. On top of the SEC column, 1 mL of embryo-conditioned medium was loaded and fractions of 1-mL eluate were collected in 16 fractions. Resulting fractions were concentrated with Amicon Ultra-2 10 k centrifugal filters (UFC201024, Merck Millipore, Billerica, MA, USA) as previously described by Pavani et al. [6] and the eluates of each fraction were retrieved from the flow-through reservoir and stored at −80 °C for further EV characterization.

### 4.6. Characterization of Extracellular Vesicles

#### 4.6.1. Western Blotting

All samples were suspended in a reducing buffer (0.005% bromophenol blue, 3% 2-mercaptoethanol, 9.2% SDS, 40% glycerol, and 0.5 M Tris-HCl (pH 6.8)) and boiled for 5 min at 95 °C. Protein samples were separated by SDS polyacrylamide gel electrophoresis and subsequently transferred to a nitrocellulose membrane (Bio-Rad, Hercules, California, USA). Next, the membrane was blocked at room temperature with 5% skim milk/BSA TBST for 45 min. Subsequently, the membranes were exposed to CD63 rabbit (1:200 in 5% BSA + 0.5% Tween PBS ab68418, Abcam, Cambridge, UK), AGO-2 rabbit (1:1000 in 5% milk + 0.5% Tween PBS, ab32381, Abcam, Cambridge, UK), and ApoA-I mouse (1:1000 in 5% milk + 0.5% Tween PBS, sc-376818, Santa Cruz, CA, USA), primary antibodies at 4 °C. After overnight incubation, the membranes were extensively washed with 0.5% Tween in PBS. Then, the membranes were incubated with the appropriate secondary antibodies (anti-mouse IgG (GE Healthcare, UK), 1:3000 in 5% milk + 0.5% Tween PBS; anti-rabbit IgG (GE Healthcare, UK), 1:4000 in 5% BSA + 0.5% Tween PBS. After a final wash step, chemiluminescence substrate (WesternBright Sirius, Advansta, Menlo Park, California, USA) was added to the membranes. Imaging was performed using Proxima 2850 Imager (IsoGen Life Sciences, De Meern, The Netherlands).

#### 4.6.2. Transmission Electron Microscopy

For transmission electron microscopy, each sample was deposited on precoated formvar/carbon support film copper mesh electron microscopy grids (FCF200H-CU-TB; Aurion, Netherlands). Grids were labeled for 45 s with 1% uranyl acetate (in double-distilled water). Prepared grids were examined using electron microscopy (JEM 1400 plus, JEOL, Benelux; and Ziess EM 109, Carl Zeiss, Jena, Germany). Moreover, images were made by Quemasa charge-coupled device camera (Olympus Soft Imaging solutions GMBH, Munster, Germany).

#### 4.6.3. Nanoparticle Tracking Analysis

Samples were analyzed by nanoparticle tracking analysis (NTA) using the NanoSight LM10 microscope (Malvern Instruments Ltd., Malvern, UK) equipped with a 455-nm laser. For each individual sample, three videos of 60 s were recorded and analyzed with detection threshold 3 and camera level 13. All videos were analyzed by NTA Software version 3.2. To achieve optimal measurements, all EV samples were diluted with PBS to obtain a particle concentration within the optimal range (3 × 10^8^–1 × 10^9^) of the NTA Software. Graphical presentations were performed using GraphPad Prism version 7.05 (GraphPad Software, San Diego, California, USA).

#### 4.6.4. EV-Track

We have submitted all relevant data of our experiments to the EV-TRACK knowledgebase (EV-TRACK ID: EV190022; Consortium et al. [24] EV-TRACK: transparent reporting and centralizing knowledge in extracellular vesicle research). The EV-METRIC score is 78%.

## Figures and Tables

**Table 1:** Comparison of extracellular vesicle isolation methods by time, price, and nanoparticle analysis.

Isolation Technique	DU	ODG	SEC		
Working Principle	EV isolation is based on sequential centrifugation steps to exclude large vesicles and cells debris and precipitate EVs	Combination of ultracentrifugation with sucrose gradient	EV isolation is based on the size difference between EVs and other particulate constituents		
Total time (h)	5	22	2		
Approximate price/sample (€)	5	15	5		
**Nanoparticle Data**	**DU EVs**	**OGD EVs (8–9 Fraction)**	**SEC EVs Fractions**		
			SEC 9	SEC 10	SEC 11
Mean	266.8 ± 2.1 nm	151.3 ± 8.8 nm	108.3 ± 6.8 nm	119.2 ± 3.5 nm	97.9 ± 2.2 nm
Mode	186.1 ± 21.9 nm	100.1 ± 20.4 nm	102.3 ± 7.0 nm	99.7 ± 6.0 nm	68.4 ± 4.0 nm
SD	91.2 ± 2.8 nm	53.9 ± 26.3 nm	34.9 ± 4.3 nm	52.4 ± 6.9 nm	39.4 ± 3.3 nm
D10	149.8 ± 2.3 nm	86.7 ± 18.9 nm	75.1 ± 7.0 nm	74.8 ± 3.6 nm	62.7 ± 4.1 nm
D50	264.5 ± 5.3 nm	127.9 ± 6.5 nm	101.1 ± 7.7 nm	104.3 ± 3.8 nm	86.4 ± 4.4 nm
D90	397.3 ± 9.1 nm	211.8 ± 39.8 nm	150.6 ± 6.9 nm	173.8 ± 8.5 nm	145.4 ± 5.8 nm
Nanoparticles/mL	3.02 ± 0.36 × 10^10^	5.50 ± 2.70 × 10^9^	2.09 ± 0.46 × 10^10^	1.50 ± 0.43 × 10^11^	1.13 ± 0.11 × 10^10^

Total time required (i.e., based on ease-of-use, turn-around time, hands-on time) and approximate cost per sample (i.e., based on the cost of centrifuge tubes and required solutions). The mode, mean value, standard deviation of size, and concentration for each EV isolation from 500 embryo culture medium method are provided. The value D50 represents the median size. Similarly, 90 percent of the distribution lies below the D90 value, and 10 percent of the population lies below the D10 value.

## Supplementary Materials

Supplementary materials can be found at https://www.mdpi.com/1422-0067/21/8/2942/s1.
