# Supplementary material for: The Separation and Characterization of Extracellular Vesicles from Medium Conditioned by Bovine Embryos
Source: Int J Mol Sci. 2020 Apr 22;21(8):2942. doi: 10.3390/ijms21082942 (PMC7215575; doi:10.3390/ijms21082942)
Supplement: Supplementary file 1 [file ijms-21-02942-s001.pdf]

Only Secondary Antibody testing

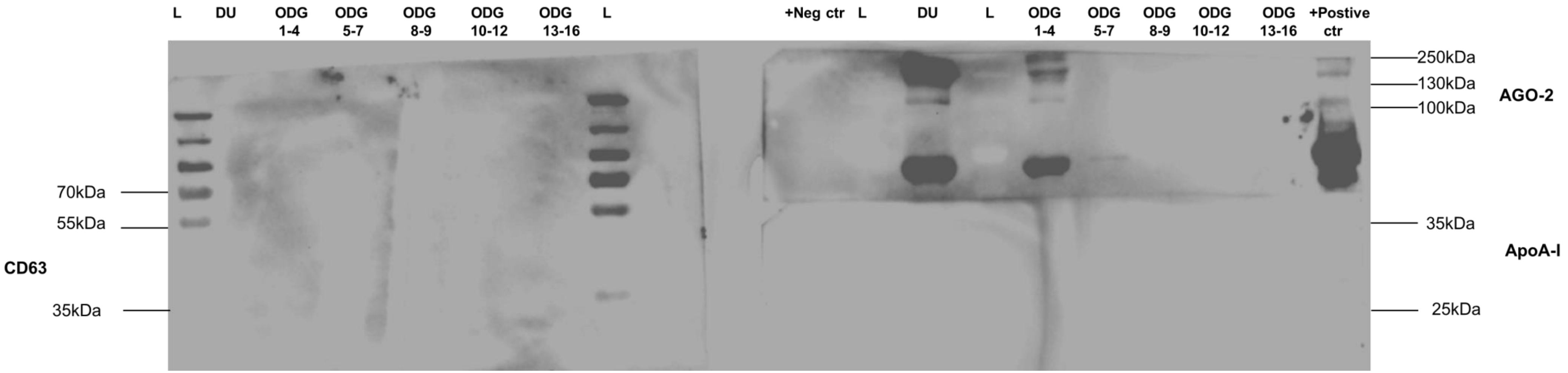

Supplementary Figure 1 Characterization of DU-EVs and ODG-EVs derived from bovine embryo conditioned medium. Western blot analysis with secondary antibodies only; anti-rabbit IgG with 5% BSA (non-reducing conditions CD63 (42 kDa)) and anti-mouse IgG with 5% milk (reducing conditions ApoA-I (28 kDa), Ago-2 (97 kDa)). Abbreviations: DU = differential ultracentrifugation; ODG 1-4 = OptiPrep density gradient fractions 1 to 4 (similar for 5-7, 8-9. 10-12, 13-16); +ctr = follicular fluid derived EVs by ODG, L = marker (protein ladder), +Neg ctr = EVs derived from Lysate HEK 293T, +Positive ctr = Plasma pure.

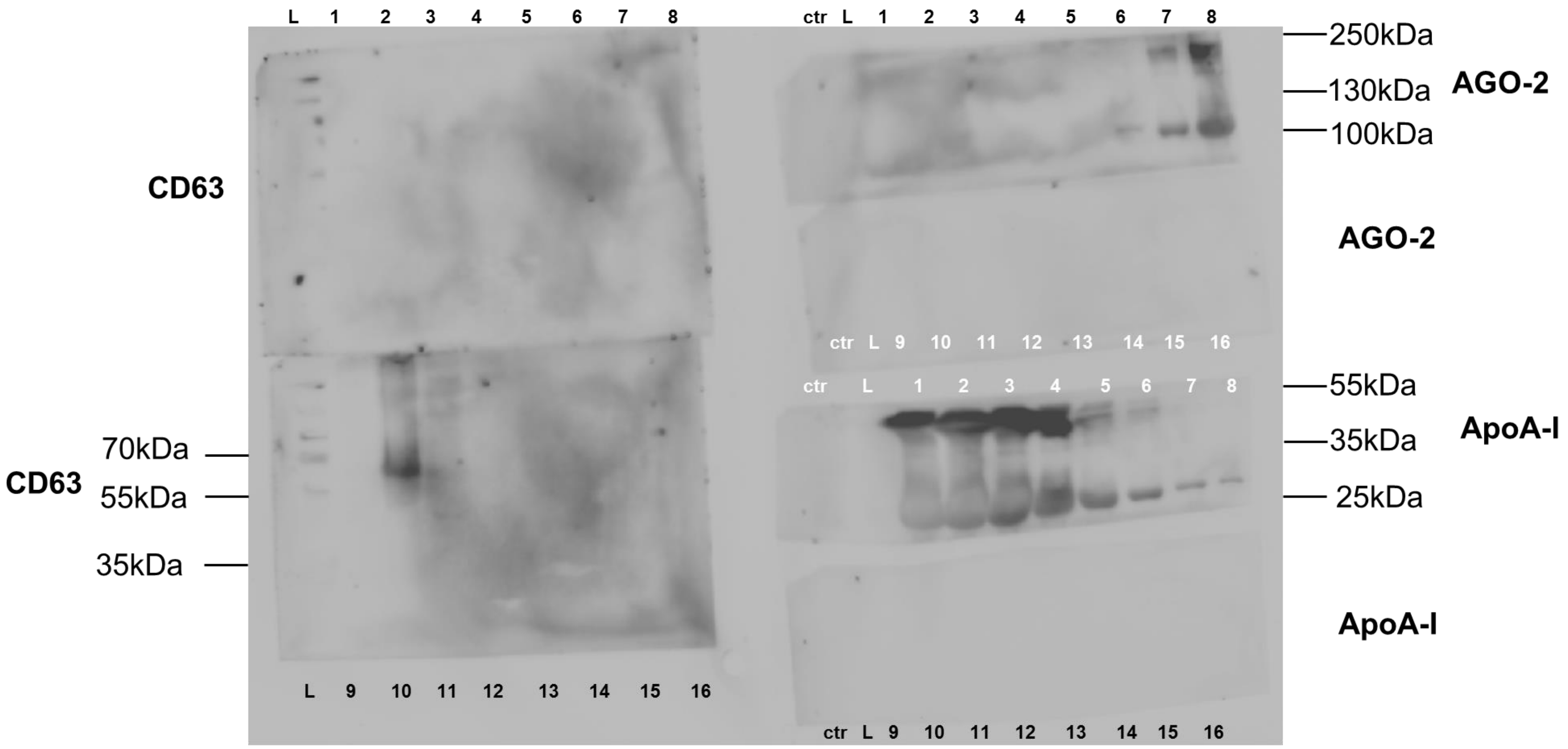

Supplementary Figure 2. Characterization of SEC-EVs derived from bovine embryo conditioned medium. Western blot analysis of only secondary antibody anti-rabbit IgG with 5% BSA (non reducing conditions CD63 (42 kDa)) and anti-mouse IgG with 5% milk (reducing conditions ApoA-I (28 kDa), Ago-2 (97 kDa)). Abbreviations: SEC 1 = size exclusion chromatography fraction 1 (similar for 2 to 16 fractions); fractions;+ctr = follicular fluid derived EVs by OptiPrep density gradient; L = marker (protein ladder).
